# Synchronous Esophageal Squamous Cell Carcinoma and Esophageal Variceal Bleeding due to Idiopathic Portal Hypertension: A Case Report

**DOI:** 10.4021/gr300e

**Published:** 2011-03-20

**Authors:** Ahmet Cumhur Dulger, Ozgur Kemik, Huseyin Begenik, Aziz Sumer, Deniz Bulut, Gulay Bulut

**Affiliations:** a Yuzuncu Yil University School of Medicine, Gastroenterology, Van, Turkey; b Yuzuncu Yil University School of Medicine, General Surgery, Van, Turkey; c Yuzuncu Yil University School of Medicine, Internal Medicine, Van, Turkey; d Yuzuncu Yil University School of Medicine, Radiology, Van, Turkey; e Yuzuncu Yil University School of Medicine, Pathology, Van, Turkey

**Keywords:** Esophageal varices, Idiopathic portal hypertension, Squamous cell cancer

## Abstract

Squamous cell carcinomas of esophagus are responsible for more than 80% of esophageal malignancies in Turkey. Idiopathic portal hypertension is a rare underlying cause of esophageal variceal bleeding. In such cases, detection of concomitant esophageal squamous cell cancer is also a rare occurrence. We report an unusual case of bleeding esophageal varices secondary to idiopathic portal hypertension associated with esophageal squamous cell cancer. To our knowledge, until now, there have been no reported cases of esophageal variceal bleeding due to idiopathic portal hypertension associated with esophageal squamous cell cancer. This case report demonstrates the two different conditions which may cause esophageal bleeding and there may be an association between idiopathic portal hypertension and esophageal squamous cell cancer.

## Introduction

Idiopathic portal hypertension (IPH) is characterized by portal hypertension, variceal bleeding, splenomegaly and the detoriation of hepatic functions in the absence of significant liver disease [1]. This syndrome was firstly described in 19th century by Banti, and it carries his name [2]. Other terms used to describe similar diseases IPH in Japan [3], non-cirrhotic portal fibrosis (NCPF) in India [4], and hepatoportal sclerosis (HPS) in the USA [5]. IPH is a leading cause of variceal bleeding in Indian subcontinent where it represents a quarter of the cases that bleed due to esophageal varices [6, 7].

The common causes of IPH are extra hepatic portal venous obstruction, non-cirrhotic portal fibrosis or idiopathic portal hypertension, schistosomiasis, primary or secondary biliary cirrhosis (pre-cirrhotic stage), congenital hepatic fibrosis, veno-occlusive disease, nodular regenerative hyperplasia, partial nodular transformation, hepatoportal sclerosis, and peliosis hepatitis. The development of esophageal variceal hemorrhage is an important event in the natural history of IPH [4].

Squomous cell cancer of esophagus (ESCC) is one of the most important and deadliest cancers worldwide as well as the Turkey. It is the sixth leading cause of death from cancer [8]. The association between ESCC and esophageal variceal bleeding secondary to IPH is very rare. There are few case reports on the association of ESCC with esophageal variceal bleeding. But most of these cases are associated with endoscopic sclerotheraphy of varices [9, 10].

Herein we describe an esophageal squamous cell cancer patient who presented with esophageal variceal bleeding related to idiopathic portal hypertension.

## Case Report

A 30-year-old woman was seen in the gastroenterology clinic of this hospital because of gastrointestinal bleeding. The patient had previously been healthy and took no medications. The patient was born in the eastern part of Turkey and was a farmer in a village.

Physical examination revealed ortostathic hypotension, melena and splenomegaly but the liver was not palpable. The remainder of the physical examination was normal. The patient’s white-cell count was 1.3 × 10^12^/L. A differential cell count revealed 50% polymorph nuclear cells, 42% bands, 4% lymphocytes, and 4% monocytes. The hemoglobin level was 9.9 g/dl, and the platelet count was 79.0 × 10^12^/L. The serum creatinine level was 0.55 mg/dl, albumin level 4.31 g/dl, aspartate aminotransferase level 16 U/L (normal range, 0 to 31), alanine aminotransferase level 16 U/L (normal range, 0 to 31), alkaline phosphatase level 206 U/L (normal range, 0 to 240), and total bilirubin level 0.25 mg/dl (normal range, 0.0 to 1.0). The international normalized ratio (INR) was 0.95.

Tests for hepatitis C antibody, hepatitis B surface antigen, hepatitis B core antibody and hepatitis D antibody were negative. Doppler ultrasonography of the abdomen revealed splenomegaly and dilated portal and splenic veins without intraluminal thrombus.

**Figure 1:**
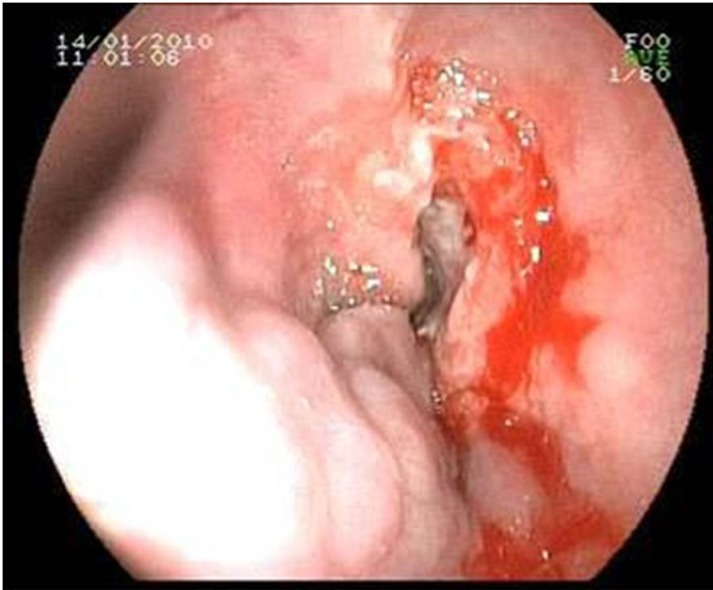
Endoscopic appearance of the esophageal mass (right) and the varices (left side).

On upper gastrointestinal endoscopy, there were four columns of varices at the distal esophagus and were treated by endoscopic varices ligation. Additionally an ulcerous mass protruding between variceal columns was seen and an endoscopic esophageal biopsy was performed (Fig. 1).

**Figure 2:**
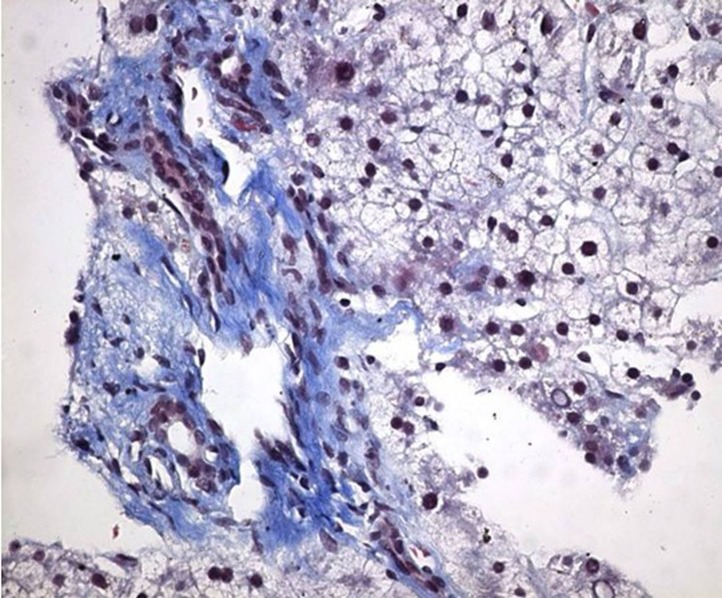
Photomicrograph of portal fibrosis and enlargement in the liver biopsy (Masson’s Trichrome stain × 400).

**Figure 3:**
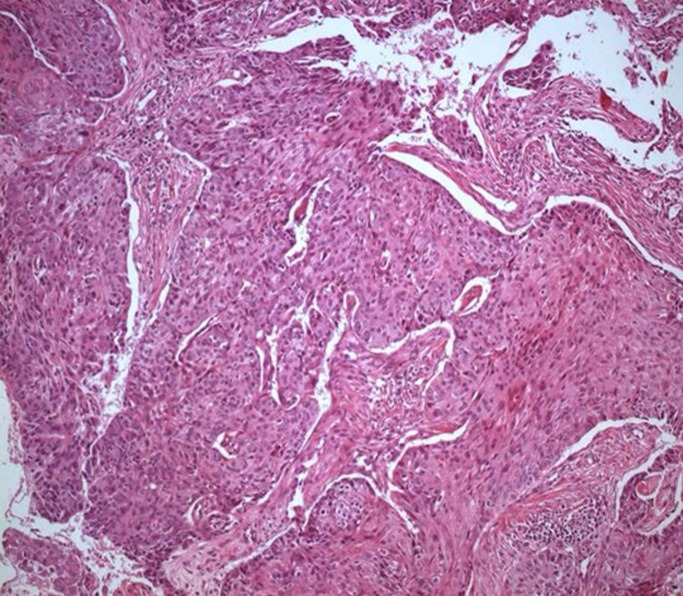
Photomicrograph of squamous cell carcinoma in the esophageal biopsy (H&E stain × 100).

**Figure 4:**
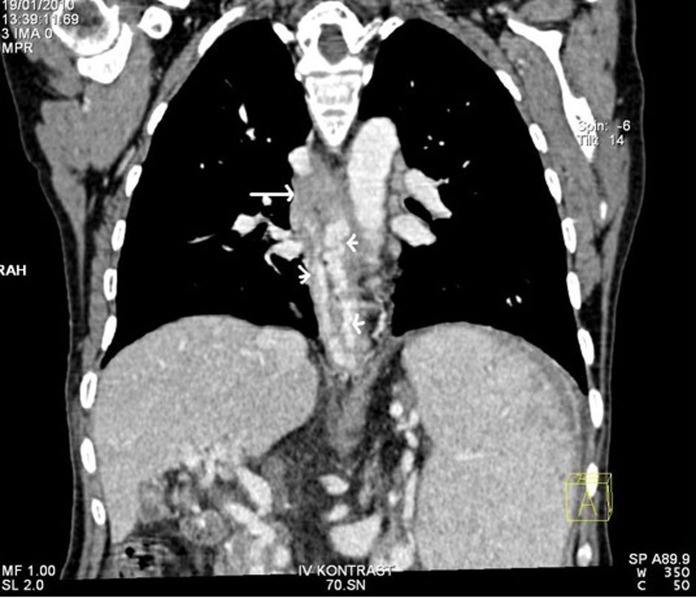
CT of the chest after the intravenous administration of contrast material confirmed the presence of esophageal mass (tall arrow) and varices (short arrows).

The liver biopsy showed portal fibrosis and sinusoidal dilatation and the esophageal biopsy revealed SCC (Fig. 2, 3). A thorax computerized tomography scan revealed the esopahageal mass and varices (Fig. 4).

The patient was diagnosed as having esophageal SCC with associated NCPF. She was transferred to a tertiary care hospital for possible esophageal surgery.

## Discussion

We have described a patient with ESCC having associated IPH. IPH associated with ESCC resulting in bleeding esophageal varices is a rare cause of hematemesis. Any of these diseases individually or in combination may be responsible for her current illness. Upper gastrointestinal bleeding is a feature of both of the portal hypertension and esophageal SCC. As presented above, the esophagogastroduodenoscopy on the emergency admission showed mid-esophageal mass superimposed on the bleeding esophageal varices. A biopsy from this mass performed during emergency admission revealed a squamous cell carcinoma.

IPH is mostly seen in Japan, India and Iran [3, 11, 12], but it has been reported from all over the world as well as Turkey [13]. The diagnosis of IPH is suggested when the biopsy specimen of the liver shows either portal tracts with fibrous enlargement or no abnormality [14].

As described above, the diagnosis of IPH was based on the following criteria: (a) no underlying factors of chronic liver disease, (b) portal hypertension and esophageal varices with normal liver function and absence of hepatic cirrhosis, (c) ultrasonographic findings of hepatosplenomegaly and portal hypertension, (d) the biopsy specimen of the patient with features of portal hypertension and moderate periportal fibrosis [4].

The majority of patients with IPH have near normal hepatic test results. Anemia, leucopenia and thrombocytopenia (hypersplenism) may be present. Prolonged prothrombine time has been detected in some IPH patients [13].

Idiopathic portal hypertension may be a coincidental finding during investigation of symptoms related to the underlying diseases, or it may present with life-threatening gastrointestinal hemorrhage as seen in this case. Only a few cases of portal hypertension coexistence with esophageal SCC have been reported in humans [15-17].

The relationship between endoscopic injection sclerotherapy and developing squamous cell carcinoma of the esophagus is well described. Recent reports have suggested that exposure to sclerosant agents may be associated with esophageal SCC particularly in patients with liver cirrhosis and It is proposed that sclerotherapy could cause the development of carcinoma of the esophagus because of mucosal injury [18].

Upper gastrointestinal cancers are important causes of morbidity and mortality all over the world as well as in Turkey. Northern part of Iran and Eastern part of Turkey are still in the Asian esophageal cancer belt [19, 20].

SCC is solely linked to a low socioeconomic status [21]. In Van region of Eastern Turkey, esophageal and gastric cancers are the most prevalent malignancies both in females and males. Turkish patients with esophageal squamous cell cancer reside generally in rural areas where socioeconomically and hygienic conditions are poor and exposure to environmental carcinogens such as aflatoxin is common [20].

As this case illustrates, bleeding esophageal varices secondary to idiopathic portal hypertension is potentially curable by endoscopic band ligation with somatostatin analogues [22]. So, we performed the endoscopic band ligation and began treatment with intravenous octreotide for the esophageal varices. Prophylaxis of infection with ceftriaxone was also started.

The standard curative surgical approach to esophageal cancer has been thoracotomy and laparotomy with extensive lymph node dissection. The role of esophageal surgery in persons with esophageal varices and ESCC has been questioned because of the risk of massive bleeding [23]. However, if a subtotal esophagectomy can be performed successfully, as it was in this patient, it could be the best way to treat her esophageal cancer.

The present case report illustrates the possibility that esophageal variceal bleeding due to IPH may be associated with ESCC. These are two different diseases which share the same etiologic factors such as lower economical status and living in rural areas of Asia. Thus, the possibility that poor environmental conditions might be one of the mechanisms driving esophageal ESCC in patients with IPH, is a particularly interesting hypothesis and these findings suggest that, as for ESCC, environmental and genetic factors may also contribute to the ethiopathogenesis of IPH.

In conclusion, ESCC can be associated with IPH, and environmental, immunological and genetic ties between ESCC and IPH may be responsible for their coexistence in our case.
